# Investigation of Antimicrobial Resistance Genes in *Listeria* *monocytogenes* from 2010 through to 2021

**DOI:** 10.3390/ijerph19095506

**Published:** 2022-05-01

**Authors:** Robert M. Hanes, Zuyi Huang

**Affiliations:** Department of Chemical Engineering, Villanova University, Villanova, PA 19085, USA; rhanes01@villanova.edu

**Keywords:** antimicrobial resistance, *Listeria monocytogenes*, listeriosis, principal component analysis, hierarchical clustering

## Abstract

Antimicrobial resistance (AMR) is a serious public health issue. Due to resistance to current antibiotics and a low rate of development of new classes of antimicrobials, AMR is a leading cause of death worldwide. *Listeria monocytogenes* is a deadly foodborne pathogen that causes listeriosis for the immunocompromised, the elderly, and pregnant women. Unfortunately, antimicrobial resistance has been reported in *L. monocytogenes*. This study conducted the first comprehensive statistical analysis of *L. monocytogenes* isolate data from the National Pathogen Detection Isolate Browser (NPDIB) to identify the trends for AMR genes in *L. monocytogenes*. Principal component analysis was firstly used to project the multi-dimensional data into two dimensions. Hierarchical clustering was then used to identify the significant AMR genes found in *L. monocytogenes* samples and to assess changes during the period from 2010 through to 2021. Statistical analysis of the data identified *fosX*, *lin*, *abc-f*, and *tet*(*M*) as the four most common AMR genes found in *L. monocytogenes*. It was determined that there was no increase in AMR genes during the studied time period. It was also observed that the number of isolates decreased from 2016 to 2020. This study establishes a baseline for the ongoing monitoring *of* *L. monocytogenes* for AMR genes.

## 1. Introduction

Antimicrobial resistance (AMR) has been a general concern since antibiotics have been in use. Sulfa drugs, discovered in 1935, were the first widely used antibiotics and today there is widespread resistance to them [1]. Fleming was concerned about the development of penicillin resistance, and it was first observed in 1940 before penicillin was in widespread use. Tetracycline was introduced in 1950 and resistance was first observed in 1959, while vancomycin was introduced in 1959 and resistance was first observed in 1979 [2]. From the 1960s to the 1980s, AMR was not a significant concern due to the development of new antimicrobials and the discovery of new classes of antibiotics [2].

Today there are more than 20 classes of antibiotics available [3], and AMR is a serious public health issue due to increasing resistance and the low rate of development of new classes of antimicrobials. It is estimated that AMR is a leading cause of death worldwide after stroke and heart disease [4]. AMR not only increases the risk of infectious diseases, but it also negatively impacts other healthcare advances due to the risk of infection during treatments. In 2019, the CDC reported nearly 3 million infections and more than 35,000 deaths due to resistant microorganisms [5]. In Europe, such infections were responsible for more than 426,000 illnesses and 33,000 deaths in 2019 [6].

The CDC estimates that there are approximately 48 million cases of foodborne illnesses per year in the United States. Listeriosis, while not common, is one of the leading causes of death from foodborne illnesses [7]. In the U.S., there are approximately 1600 infections per year that result in about 260 deaths, corresponding to a hospitalization rate of 94% and a mortality rate of 16% [8]. The fatality rate can be as high as 30% in immunocompromised people, the elderly, and pregnant women [9]. *Listeria monocytogenes*, the pathogen that causes listeriosis, has the third highest mortality of foodborne pathogens in the U.S. [10]. It is part of the genus *Listeria*, which contains seven species; however, it is the only species pathogenic to animals and humans. *L. monocytogenes* has been found to be a highly occurring pathogen in several countries, including the United States, United Kingdom, Australia, Canada, and Mexico [11]. It is found in the environment and is carried by animals [12]. Humans are infected with the bacteria primarily by eating or handling contaminated food or touching contaminated surfaces [12]. It can also be transmitted from mother to child in utero or at birth [12]. *L. monocytogenes* is susceptible to a wide range of antibiotics active against Gram-positive bacteria, except cephalosporins and fosfomycin, for which it has inherent resistance [13]. The most common treatment for listeriosis is ampicillin used alone or in conjunction with gentamicin [13].

The first resistant strains of *L. monocytogenes* were isolated in France in 1988, including the first multidrug resistant strain [13]. Up through 1999, only sporadic resistance had been observed in antibiotics, including tetracycline, chloramphenicol, erythromycin, and streptomycin [13]. A foodborne strain was found to be resistant to trimethoprim, part of a secondary treatment for listeriosis for patients who are allergic to penicillins [13]. Notably, resistance to penicillins and gentamicin was not observed [13]. Similar trends were observed in a study of AMR in *L. monocytogenes* strains isolated in France between 1926 and 2007. That study also confirmed the presence of resistance genes and compared the minimum inhibitory concentrations (MICs) for various antibiotics. It was found that MICs from 1989–2007 increased compared to 1926–1988 [14].

In a study published in 2001, antimicrobial susceptibility testing (AST) was performed on *Listeria* isolates from retail foods purchased in the greater Dublin area. Resistance to penicillin and ampicillin was observed in 3.73% and 1.98% of 1001 isolates, respectively [15]. These were the second and third highest percentages of resistance observed, after tetracycline resistance, which was observed in 6.3% of isolates [15]. AST of 317 *L. monocytogenes* isolates collected from food, humans, and the environment in Italy between 1998 and 2009 found resistance to ampicillin, penicillin, gentamicin, and trimethoprim-sulfamethoxazole in 100% of the isolates [16]. That study also found that there was an increase in resistance in isolates from 2007–2009 compared to isolates analyzed from 1998–2006 [16].

In a 2014 study of *L. monocytogenes* from meat products and processing environments, resistance was observed in 34.5% of 206 isolates. The highest resistance was to oxacillin. There was low resistance to tetracycline and no resistance to penicillin [17]. Additionally, in a recent study in Uruguay, 50 *L. monocytogenes* isolates from various sources were subject to AST and analyzed for AMR genes. All of the samples were determined to be fully susceptible to penicillin, gentamicin, and trimethoprim-sulfamethoxazole [18]. This study also found that all *L. monocytogenes* isolates contained the resistance genes *fosX* and *lin* [18].

In a review published in 2021 and focused on foodborne pathogens isolated from dairy cattle and poultry manure in Northeastern Ohio, *L. monocytogenes* was the second highest pathogen found in the isolates. All the isolates were resistant to at least one of the antibiotics tested. Significantly, 89.5% of the 67 *L. monocytogenes* isolates were found to have ampicillin resistance and 47% were found to have penicillin resistance [19].

These studies show a trend of increasing AMR observed in *L. monocytogenes*. While resistance to antibiotics used in the first-line treatment used for listeriosis has not yet been widely reported in humans, resistance has been reported in animals, as discussed in the studies cited above. This is concerning because resistance in animals could eventually be transferred to humans. These observations demonstrate the importance of continued surveillance of AMR in *L. monocytogenes* and the need for the development of other therapeutic options. A potential method for monitoring the development of AMR is to monitor the occurrence of AMR genes.

An active surveillance program is critical to monitoring the development of AMR among pathogens. In the United States, the National Center for Biotechnology Information (NCBI) (Bethesda, MD, USA) maintains the National Database of Antibiotic Resistant Organisms (NDARO) to aid surveillance of pathogens [20]. As part of this effort, the NCBI Pathogen Detection Isolate Browser (NPDIB) was developed to identify AMR genes observed in pathogen bacterial genomic sequences [21]. The NPDIB consists of the Reference Gene Catalog and AMRFinderPlus. The Reference Gene Catalog includes sequences from food, the environment, and patients received from public health agencies around the world. AMRFinderPlus is a program that identifies the AMR genes in both protein and nucleotide sequences that have been submitted to the Reference Gene Catalog. AMRFinderPlus has been validated against two different datasets [22]. While the data from the NPDIB data have been used to determine common AMR genes for general pathogens as a whole, few studies have been conducted for the deadly *L. monocytogenes*. In this work, a statistical analysis of isolate data from the NPDIB was thus performed to: (1) identify the major AMR genes found in *L. monocytogenes* samples; (2) assess changes over time to study the occurrence and development of AMR genes in *L. monocytogenes*; and (3) establish a baseline for ongoing monitoring. The collection date and location were determined from previous work in which it was found that the NPDIB was more widely used from 2010 onwards. In this previous work, it was also found that the locations in which the *L. monocytogenes* is highly occurring are United States, United Kingdom, Australia, Canada, and Mexico [11]. Therefore, this study is focused on the NPDIB data from 2010 to 2021 for several regions in the world with data available. These regions include: Australia/New Zealand, Asia, Europe, North America, South Africa, and United Kingdom/Ireland.

## 2. Materials and Methods

### 2.1. Antimicrobial Resistance Data from the NCBI Pathogen Detection Isolate Browser

The NCBI Pathogen Detection Isolate Browser (NPDIB) currently contains almost a million total isolates covering 34 organism groups [21]. The data for this analysis were downloaded from the NPDIB on 24 February 2022, using the following search criteria:

- Organism group = Listeria monocytogenes;
- Collection date = from: 31 December 2009, to: 31 December 2021.

For this analysis, the NPDIB data were downloaded into a Microsoft Excel worksheet. The data were then organized into a matrix where each row corresponded to a *L. monocytogenes* sample with the columns including the following: scientific name, collection date, location, isolation type, serovar, and AMR genotype. As for the isolation type, the clinical category represents samples isolated from human sources, while the environmental/other category represents samples isolated from all other sources including the environment, animals, and food. Table 1 shows a detailed description of each column category.

After the data were downloaded from the NPDIB, they were formatted for subsequent analysis. A MATLAB program was written to extract the AMR genotype data from a single column into multiple columns. As downloaded from NPDIB, the AMR genotype data are in the following format: “aac(6′)-I = COMPLETE, abc-f = COMPLETE, fosX = COMPLETE, lin = COMPLETE, msr(C) = COMPLETE”. These data were processed to create one column per gene, populated with a 1 if the gene was found in the sample and 0 if the gene was not found. The data in the other columns were manipulated to align the formats and to replace text entries with numerical entries. Table 2 summarizes the data in the final spreadsheet that was used for analysis.

### 2.2. Principal Component Analysis and Hierarchical Clustering

The data from the NPDIB were downloaded as described above. The data matrix contains a total of 35,753 rows, each of which corresponds to an isolate sample of *L. monocytogenes* entered from January 2010 through December 2021. These samples were further analyzed to obtain a matrix in which each row represents one gene while each column represents one year with the detection occurrence of the gene in the corresponding year recorded in the matrix cell. The data contained a total of 65 AMR genes for 2010 to 2021 resulting in a matrix that contains 65 rows and 12 columns.

Due to the number of dimensions, these data were analyzed using principal component analysis (PCA) and hierarchical clustering (HC) to identify the highly occurring AMR genes by region and setting. PCA allows the visualization of multi-dimensional data in two dimensions. The data are expressed in terms of new variables that are linear combinations of the existing variables. The principal components PC1 and PC2 are those that retain the most variation from the original data [23]. In this work, PCA is used to project AMR genes into the PC1~PC2 two-dimensional space so that the outlier genes, which typically show higher occurrence over years, are identified for further investigation. While AMR genes can be visualized in PCA, certain genes are lumped together. PCA does not directly provide the correlation relationship between individual AMR genes. Therefore, hierarchical clustering is further used to group the genes projected onto the PC1~PC2 space into clusters that are similar to each other in the format of dendrograms. PCA and HC were performed, and graphs were generated using the free statistical software package R, version 1.4.1106, implemented in RStudio (Boston, MA, USA) [24].

## 3. Results

### 3.1. Occurrence of Listeria monocytogenes

**Figure 1:**
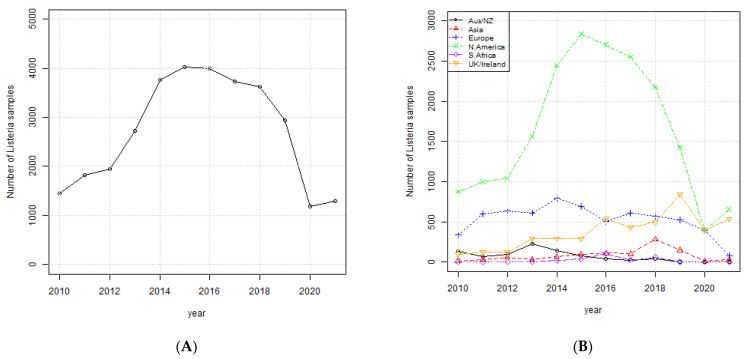
(**A**) Total number of *L. monocytogenes* samples per year; (**B**) *L. monocytogenes* samples per region per year.

The data matrix generated from the NPDIB data included 35,753 *Listeria monocytogenes* isolates from the six regions identified above: Australia/New Zealand, Asia, Europe, North America, South Africa, and UK/Ireland. In Figure 1A, the total number of samples peaked in 2016 and then declined during the subsequent years. Figure 1B shows this trend is driven by North America, consisting of USA, Canada, and Mexico, where *L. monocytogenes* is most prevalent. In UK/Ireland, the occurrence of *L. monocytogenes* increased modestly over the time period. In the other four regions, the occurrence of *L. monocytogenes* was relatively constant over the time period. In all cases, the number of samples declined in 2020 and 2021.

Lower numbers of samples were expected in 2020 and 2021, likely a result of public health measures enacted due to the COVID-19 pandemic. The decline in the number of samples from 2016 through 2019 could potentially be due to an actual decrease in the occurrence of *L. monocytogenes* or simply due to fewer samples being submitted to the NPDIB.

**Figure 2:**
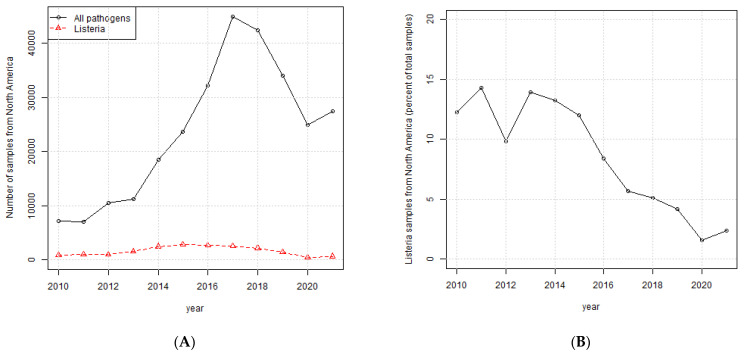
(**A**) Total number of samples from all pathogens *and L. monocytogenes* per year; (**B**) *L. monocytogenes* samples as a percentage of total pathogens per year.

The decline in the number of samples of *L. monocytogenes* from 2016 through 2019 was further investigated by determining the total number of samples submitted to NPDIB per year from North America for all pathogens. Figure 2A indicates that the total number of samples submitted per year from North America for all pathogens shows a similar trend as *L. monocytogenes* samples. However, Figure 2B shows the percentage of *L. monocytogenes* samples declined over the time period starting in 2013. This suggests that the decrease in *L. monocytogenes* samples is due to a lower prevalence of the pathogen in North America and not simply due to an overall lower number of samples being collected and submitted to the NPDIB.

### 3.2. Presence of Antimicrobial Resistance Genes

A comprehensive analysis of *Listeria monocytogenes* isolates was performed to determine the highly occurring AMR genes and to determine if AMR genes increased over time. For the time period 2010 to 2021, there are 35,753 samples for which the organism group is identified as *L. monocytogenes*. There were a total of 65 AMR genes found in these samples. Multivariate statistical analysis, mainly PCA and hierarchical clustering, was performed and the highly occurring genes were identified by region and isolation type.

**Figure 3:**
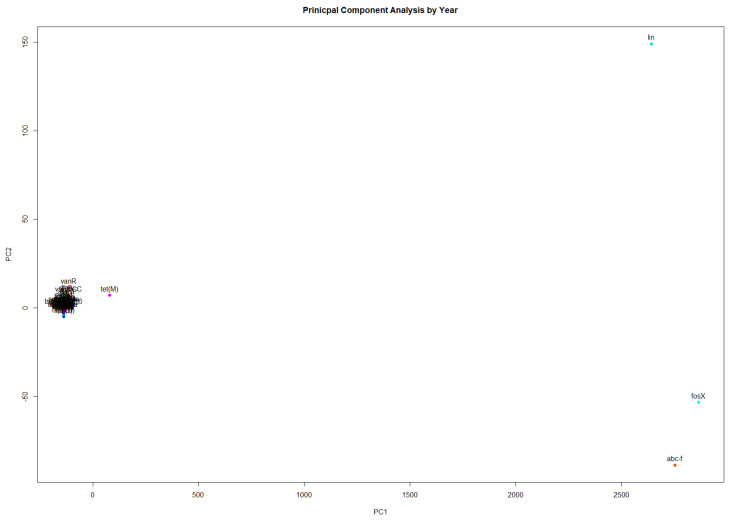
PCA to identify the most frequently occurring AMR genes in *L. monocytogenes* samples.

Upon initial review of the data, it was found that the genes *fosX* and *lin* are present in nearly every sample. The gene *fosX* is present in 99.98% of the samples and *lin* is present in 97.8% of the samples. The following analysis was performed using samples that contained at least one AMR gene other than *fosX* or *lin*. This resulted in a modified data matrix of 10,039 samples, in which reach row represented a *L. monocytogenes* sample, each column represented a gene, and the entries represented if the gene was detected in the sample. PCA was performed to determine the highly occurring genes. Figure 3 shows that *fosX*, *lin*, and *abc-f* are the most frequently occurring genes, followed by *tet(M)* and *vanC*, *vanR*, *vanS*, *vanT*, and *vanXY-C*, which occur at a greater frequency than the remaining 56 AMR genes.

**Figure 4:**
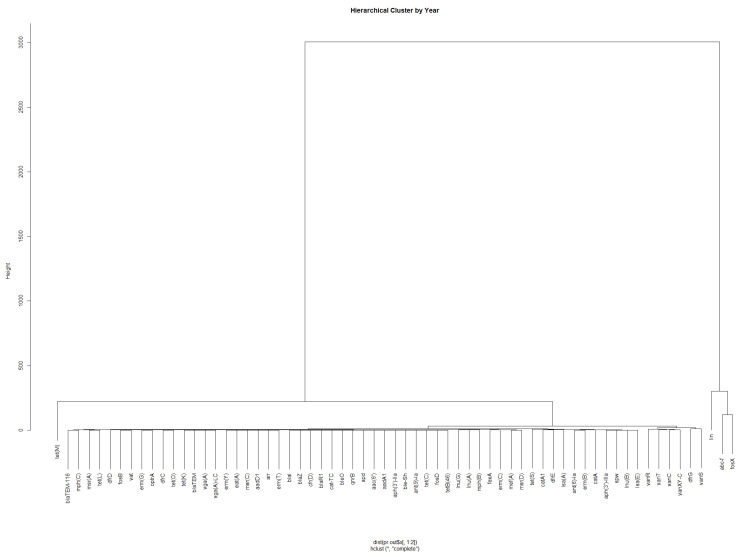
Hierarchical clustering to identify the AMR genes that occurred with similar frequency in *L. monocytogenes* samples.

Hierarchical clustering (HC) was also performed to identify clusters of genes that occurred with similar frequencies. Figure 4 shows that *fosX*, *abc-f*, and *lin* are clustered together, followed by *tet(M)* and the vancomycin resistance genes *vanC*, *vanR*, *van*S, *vanT*, and *vanXY-C*. The gene *dfrG* was also clustered with the vancomycin resistance genes.

**Figure 5:**
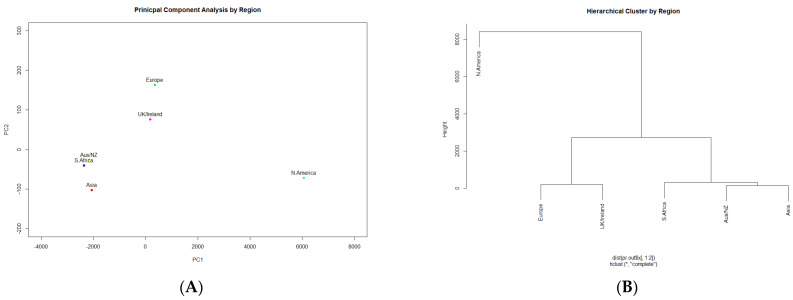
PCA (**A**) of regions on the basis of AMR genes; HC (**B**) of regions on the basis of AMR genes.

PCA and hierarchical clustering were also performed to compare regions based on the occurrence of AMR genes. For this analysis, a data matrix was generated in which each column represented the region from which the sample was collected and there was one column per gene in which the entries represented if the gene was detected in the region for each row. This resulted in a modified data matrix of 32,509 samples as there were a number of samples for which the location was not identified. The PCA and HC results are shown in Figure 5, which indicate that similar resistance genes were observed in the following clusters: (1) North America, (2) Europe and UK/Ireland, and (3) Asia, Australia/New Zealand, and South Africa. These results are not unexpected because the regions are clustered by geographical proximity.

Table 3 lists the highly occurring genes found in each region. In all cases except Asia and North America, *fosX*, *lin*, and *abc-f* represent greater than or equal to 97% of the AMR genes observed. For North America, they represent 93% of the observed genes and for Asia, they represent 72% of the observed genes.

Table 4 lists the highly occurring genes by isolation type. In both cases, the genes listed represent 99.5% of the genes observed in each setting. It is expected to find more variety in AMR genes in the environmental/other category because these represent a wider variety of sources and typically AMR genes originate in these sources before being transferred to humans.

### 3.3. Investigation of Highly Occurring AMR Genes

**Figure 6:**
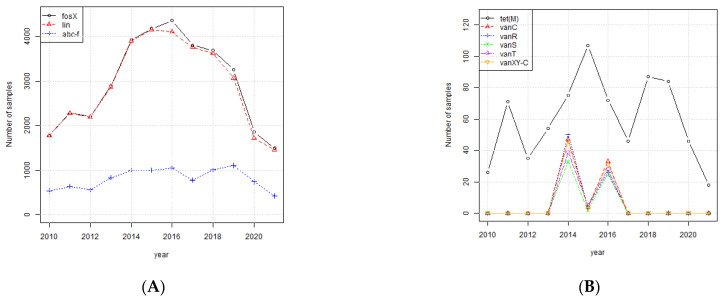
(**A**) Number of *L. monocytogenes* samples per year with genes *fosX*, *lin,* and *abc-f* and *tet(M)*. (**B**) Number of *L. monocytogenes* samples per year with genes *vanC*, *vanR*, *van*S, *vanT*, and *vanXY-C*.

Figure 6A shows the number of samples of *Listeria monocytogenes* with genes *fosX*, *lin, abc-f,* and *tet(M)* and the second chart (b) shows the number of samples with *vanC*, *vanR*, *van*S, *vanT*, and *vanXY-C*. The genes *fosX* and *lin* show the same trend as the number of samples because they are present in nearly all the samples. The gene *abc-f* is relatively constant from 2013 through to 2019, suggesting that the frequency of this gene is increasing since the number of samples is declining over the time period. The gene *tet(M)* shows a similar trend to the number of samples suggesting the frequency of this gene remained constant over the time period. The five vancomycin resistance genes (*vanC*, *vanR*, *van*S, *vanT*, and *vanXY-C)* spiked in 2014 and 2016 but have not been observed in recent years.

**Figure 7:**
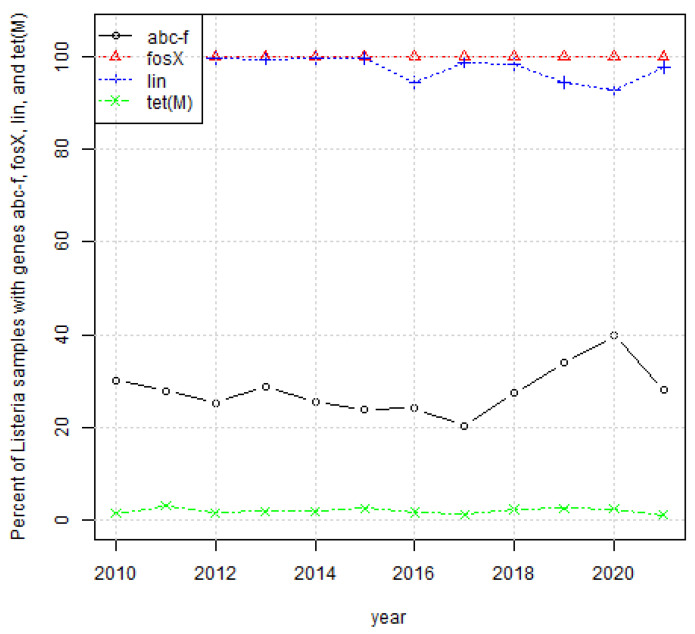
Percentage of *L. monocytogenes* samples per year with genes *fosX*, *lin, abc-f,* and *tet(M)*.

Figure 7 confirms that the percentages of samples containing genes *fosX*, *lin*, and *tet(M)* fluctuated around the same values for samples from 2010 through to 2021. However, there was an increase in the frequency of *abc-f* from 2017 through to 2020.

### 3.4. The Biological Functions of the Highly Occurring Genes

The four highest occurring genes were *fosX*, *lin*, *abc-f,* and *tet(M)*. The *fosX* and *lin* AMR genes are present in nearly all samples. These genes impart antimicrobial resistance to fosfomycin, quinolones, and expanded-spectrum cephalosporins [18]. The *abc-f* gene is a lincomycin resistance gene [25] and the *tet(M)* gene is a tetracycline resistance gene [26]. Table 5 summarizes the biological functions of all the highly occurring AMR genes.

## 4. Discussion

The results presented in Section 3.1 indicate that the frequency of *L. monocytogenes* has been declining in North America since 2015. To corroborate this result, the number of *Listeria* infections in the United States was reviewed from two other sources: the Foodborne Diseases Active Surveillance Network (FoodNet) and NORS. These two sources support the conclusion from the NPDIB data that *L. monocytogenes* did not increase from 2010 through to 2021.

FoodNet tracks infections commonly transmitted through food since 1996. FoodNet’s surveillance area includes 15% of the US population across 10 states [39]. Table 6 shows the results for *Listeria* from the pathogen surveillance tool from 2010 through to 2020 [39]. These results show a more consistent rate of *Listeria* infections with a small peak in 2017. However, it is noted that the FoodNet data are smaller datasets over a small segment of the United States and the results support the general observation that *Listeria* infections are not increasing over time.

The National Outbreak Reporting System (NORS) reports outbreaks for foodborne disease per year. Table 7 shows the results reported by NORS for *Listeria* from 2010 through to 2018 [40]. Similar to the FoodNet data, the NORS data do not align exactly with the trend seen in the NPDIB results, but they also support the general observation that *Listeria* infections are not increasing over time.

From the results in Section 3.2, it is noted that a diversity of AMR genes was not observed in *L. monocytogenes* during the time period. There are two AMR genes, *fosX* and *lin*, that were present in nearly all samples. The *fosX* gene has been demonstrated to be part of the core genome of *L. monocytogenes* [41]. There are two additional AMR genes, *abc-f* and *tet(M)*, that occurred at higher frequency. The frequency of occurrence of the gene *tet(M)* did not change over time and is consistent with observations of tetracycline resistance in the works cited in Section 1. The frequency of occurrence of the gene *abc-f* increased from 2017 through to 2020 but decreased in 2021. Based on the information included in Table 5, this gene does not confer resistance to the primary antibiotics used to treat listeriosis.

There are five AMR genes, *vanC*, *vanR*, *vanS*, *vanT*, and *vanXY-C*, that occurred at a lower frequency. These were observed in the period 2014 to 2016 and have not been observed since. It is interesting to note that these genes were present only in environmental/other isolates and only in the years that *L. monocytogenes* had the highest frequency of occurrence. Going forward, isolates in the NPDIB should be monitored closely to see if these AMR genes occur again as that could potentially be an indicator of increased AMR and a rise in frequency of *L. monocytogenes*.

The current treatment regimen for serious cases of listeriosis is ampicillin either used alone or in conjunction with gentamicin. The findings in this analysis are important because they demonstrate that AMR genes that confer resistance against ampicillin and gentamicin are not widely present in *L. monocytogenes* at this time. For example, the AMR gene *ampR*, which confers ampicillin resistance, and the AMR genes *aac(6′)* and *aph(2″)*, which confer gentamicin resistance, were not found in any of the *L. monocytogenes* isolates. This indicates that the current treatment regimen will continue to be medically relevant. However, the results from NPDIB are in contrast with more recent studies cited in Section 1 in which resistance to penicillin, ampicillin, and gentamicin has been observed, e.g., [15,16,19]. As a result, ongoing monitoring of AMR genes in *L. monocytogenes* in the environment, animals, and humans is important because tracking the occurrence of genes that impart resistance to the first-line antibiotics will improve understanding of the future risks of the effectiveness of these treatments. In addition, the highly occurring genes can guide research for new treatments against *L. monocytogenes*. These AMR genes serve as potential drug targets for new and alternative treatments, for example, new antibiotics or compounds that will work synergistically with current antibiotics to treat resistant *L. monocytogenes* infections.

Overall, these results demonstrate that the AMR genes present in *L. monocytogenes* samples from six regions are not changing over time and AMR resistance genes that impact the current treatment regimen were not observed in the NPDIB data. Although the observations from the NPDIB data are conclusive and generally supported by other sources, there are a couple of questions regarding the NPDIB data. First, the database is incomplete in several ways. For example, metadata are either missing or not consistently formatted, and there are very little data for antibiotic susceptibility testing. Second, the total number of samples reported in the NPDIB for all countries, all organisms declined from 2018 to 2019 and declined in the United States from 2017 to 2019. It is not clear if these declines are due to lower frequencies of isolates or due to lower compliance in submitting samples. Lower compliance in submitting samples to the NPDIB could potentially impact the validity of conclusions made from analysis of NPDIB data. For example, it may affect how representative the data are if not all health agencies in the USA or internationally are not uniformly collecting and sequencing samples and submitting to NCBI for analysis and inclusion in the NPDIB.

## 5. Conclusions

It is concluded from the NPDIB data that there was no increase in antimicrobial resistance genes in *L. monocytogenes* during the time period from 2010 through to 2021. This is supported by the fact that AMR genes, with the exception of *fosX* and *lin*, are not observed in a significant number of samples over the time period and by the fact that *L. monocytogenes* isolates are observed to be decreasing over time. It would be expected that an increase in antimicrobial resistance in *L. monocytogenes* would also result in an increase in the number of reported isolates.

Going forward, efforts should focus to ensure samples are submitted to NCBI and to improve the consistency in metadata because, as shown in this work, the database can be used for the surveillance of antimicrobial resistance for the 34 pathogens included in the database. *L. monocytogenes* isolates should be monitored closely for any changes in AMR genes, as well as for the appearance of ampicillin or gentamicin resistance genes. It would be critical to track and trace these cases closely as they could potentially be indicators of a rise in the frequency of *L. monocytogenes*.

## Figures and Tables

**Table 1:** Description of data downloaded from NPDIB.

Category	Description
Scientific name	*Listeria monocytogenes*
Collection date	Date the sample was collected
Location	Location from which the sample was collected
Isolation type	Clinical or environmental/other
Serovar	Serovar
AMR genotype	List of the AMR genotypes identified in sample

**Table 2:** Description of processed NPDIB data matrix used for analysis.

Category	Abbreviated Name	Entries	Comments
Scientific name	Sci_name	1 = *Listeria monocytogenes*	
Collection date	Year	2010 through to 2021	
Location	Region	1 = Australia/New Zealand 2 = Asia 3 = Europe 4 = North America 5 = South Africa 6 = United Kingdom/Ireland	
Isolation type	Epi_type	1 = clinical 2 = environmental	
Serovar	Serovar	1 = 1/2a 2 = 1/2b 3 = 4b	
AMR gene	Gene name, e.g., fosX	0 = not found in sample 1 = found in sample	There is 1 column for each gene

**Table 3:** Highly occurring AMR genes by region.

Aus/NZ	Asia	Europe	N. America	S. Africa	UK/Ireland
*fosX*	*fosX*	*fosX*	*fosX*	*fosX*	*fosX*
*abc-f*	*lin*	*abc-f*	*abc-f*	*lin*	*abc-f*
*lin*	*abc-f*	*lin*	*lin*	*abc-f*	*lin*
*erm(G)*	*tet(M)*	*tet(M)*	*tet(M)*	*fexA*	*tet(M)*
*tet(M)*	*dfrG*		*vanC*	*tet(M)*	
	*tet(S)*		*vanR*	*dfrG*	
	*ant(6)-Ia*		*vanXY-C*	*tet(S)*	
	*erm(B)*		*vanT*		
	*aph(3′)-IIIa*		*vanS*		
	*lnu(B)*		*tet(S)*		
	*spw*		*catA1*		
	*catA*				
	*catA1*				
	*mef(A)*				
	*msr(D)*				
	*erm(C)*				
	*fexA*				
	*lnu(A)*				

**Table 4:** Highly occurring AMR genes by isolation type.

Clinical	Environmental/Other
*fosX*	*fosX*
*lin*	*abc-f*
*abc-f*	*lin*
*tet(M)*	*tet(M)*
*catA1*	*vanC*
*catA*	*vanR*
*mef(A)*	*vanXY-C*
*msr(D)*	*vanT*
*dfrG*	*vanS*
*fexA*	*tet(S)*
	*dfrE*
	*fexA*
	*dfrG*
	*erm(B)*
	*lnu(G)*
	*blaTEM-116*
	*erm(C)*
	*lsa(A)*
	*mph(B)*
	*tet(L)*

**Table 5:** Biological functions of highly occurring AMR genes.

AMR Gene	Biological Function	References
*fosX*	Catalyzes hydration of fosfomycin breaking the oxirane ring	[18]
*abc-f*	ATP-binding cassette protein that mediates resistance to a broad array of antibiotic classes that target the ribosome of Gram-positive pathogens	[25]
*lin*	Ribosomal protection protein, lincomycin	[18]
*tet(M)*	Tetracycline resistance (ribosome protection), class M	[27,28]
*vanC*	Glycopeptide resistance gene; vancomycin, class C	[29]
*vanR*	Glycopeptide resistance gene; vancomycin, class R	[30]
*vanXY-C*	Glycopeptide resistance gene; vancomycin	[31]
*vanT*	Glycopeptide resistance gene; vancomycin, class T	[32]
*vanS*	Glycopeptide resistance gene; vancomycin, class S	[30]
*tet(S)*	Tetracycline resistance (ribosome protection), class S	[27,28]
*dfrE*	Trimethoprim resistance	[33]
*fexA*	Active efflux, phenicols	[28]
*dfrG*	Trimethoprim resistance	[34]
*erm(B)*	Ribosome modification-mediated resistance; macrolide, lincosamide, and streptogramin B	[27]
*lnu(G)*	Enzymatic inactivation by nucleotidylation, lincomycin	[35]
*blaTEM-116*	Β-lactamase, broad-spectrum cephalosporin	[36]
*erm(C)*	Ribosome modification-mediated resistance; macrolide, lincosamide, and streptogramin B	[28]
*lsa(A)*	Lincosamide and streptogramin A resistance	[37]
*mph(B)*	Encode phosphotransferases conferring macrolide resistance	[38]
*tet(L)*	Tetracycline resistance (active efflux), class L	[27,28]

**Table 6:** FoodNet data for *Listeria* infections by year.

Year	Infections (Incidence Per 100,000 Population)
2010	0.26
2011	0.28
2012	0.26
2013	0.25
2014	0.24
2015	0.25
2016	0.26
2017	0.32
2018	0.26
2019	0.27
2020	0.2

**Table 7:** NORS outbreak data for *Listeria* per year.

Year	Outbreaks	Illnesses	Hospitalizations	Deaths
2009	4	35	18	0
2010	5	32	29	9
2011	6	209	184	39
2012	5	41	38	6
2013	10	86	77	16
2014	14	84	79	20
2015	6	75	61	7
2016	6	77	69	10
2017	11	54	47	7
2018	4	43	38	4

## Data Availability

Not applicable.
